# Construction and characterization of a nanopore derived from the transmembrane domain of a trimeric autotransporter adhesin

**DOI:** 10.3389/fbioe.2026.1764864

**Published:** 2026-02-20

**Authors:** Jun Sasahara, Shogo Yoshimoto, Zugui Peng, Taehyun Hwang, Iori Kobayashi, Ryuji Kawano, Katsutoshi Hori

**Affiliations:** 1 Department of Biomolecular Engineering, Graduate School of Engineering, Nagoya University, Nagoya, Aichi, Japan; 2 Department of Biotechnology and Life Science, Tokyo University of Agriculture and Technology (TUAT), Tokyo, Japan

**Keywords:** electrophysiological measurement, molecular dynamics (MD) simulation, nanopore, trimeric autotransporter adhesin (TAA), type Vc secretion system

## Abstract

Bacterial secretion systems (SSs) are increasingly recognized as biological nanopores with potential biotechnological applications. Here, we engineered the transmembrane β-barrel of a trimeric autotransporter adhesin (TAA) secreted by the type Vc SS. The coiled-coil segment that occupies the central lumen of the transmembrane β-barrel of an *Acinetobacter* TAA, AtaA, was removed to design an open β-barrel pore, termed AtaApore. Polypeptides of AtaApore were produced using a cell-free expression system and reconstituted into lipid membranes. Electrophysiological measurements showed ion channel activity of AtaApore with a median conductance of 0.17 nS. Molecular dynamics simulations revealed ion transport properties, including transient trapping of Cl^−^ ions at a constriction formed by R3597 and R3622. Together, to our knowledge, these results provide the first characterization of a nanopore derived from a TAA secreted by the type Vc SS. AtaApore provides a new scaffold for nanopore engineering and a simplified model for probing the mechanism of the type Vc SS.

## 1 Introduction

Protein secretion across the outer membrane is a fundamental process in Gram-negative bacteria. By exporting proteins to the cell surface or extracellular environment, bacteria obtain nutrients, interact with their surroundings, and establish multicellular structures such as biofilms (Costa et al., 2015; Green and Mecsas, 2016). Secreted proteins also function as major virulence factors in pathogenic species (Leyton et al., 2012). Beyond these biological roles, secretion systems are increasingly recognized as biological nanopores with potential for biotechnological applications. For instance, CsgG protein, derived from the curli secretion system of *Escherichia coli*, is utilized for DNA nanopore sequencing (Goyal et al., 2014; Van der Verren et al., 2020). Furthermore, pores derived from bacterial secretion systems and other biological channels have been adapted for nanoscale sensing, including the single-molecule detection of RNA and peptides (Piguet et al., 2018; Cao et al., 2019). More recent advances have further extended biological nanopores toward peptide and protein analysis, enabling single-molecule protein reading and bringing nanopore-based protein sequencing closer to practical realization (Motone et al., 2024; Lu et al., 2025; Miyagi et al., 2025).

Among the bacterial secretion systems (SS), the type V SS is unique because the substrate protein itself carries all components necessary for translocation (Lin et al., 2002; Leo et al., 2012; Rollauer et al., 2015). The type V SS proteins are classified into subclasses Va to Ve based on structural and mechanistic features (Leo et al., 2012). The pore-forming activities and molecular characteristics of the type Va SS, such as NalP from *Neisseria meningitidis,* Hbp from *Escherichia coli*, and IgA protease from *Neisseria gonorrhoeae,* have been extensively studied (Veiga et al., 2002; Oomen et al., 2004; Roussel-Jazédé et al., 2011). The type Vb SS, such as FhaC from *Bordetella pertussis* and HMW1B from *Haemophilus influenzae* have also been characterized, and their channel properties have been analyzed using electrophysiological approaches (Méli et al., 2006; Duret et al., 2008; Fan et al., 2012). These studies established the type Va and Vb SS as valuable models for the elucidation of the autotransporter mechanism and made them promising candidates for nanopore sensors.

Trimeric autotransporter adhesins (TAAs), which belong to the type Vc SS, form a homotrimeric structure with an N-terminal passenger domain that mediates biological functions and a C-terminal β-barrel transmembrane domain that transports and anchors the passenger domain to the outer membrane (Linke et al., 2006; Bentancor et al., 2012). TAAs assemble a β-barrel in which four β-strands from each subunit together form a 12-stranded transmembrane pore that surrounds a central lumen (Meng et al., 2006). The threefold-symmetric architecture of the barrel makes a narrow hydrophilic environment, and the lumen is normally occupied by an elongated coiled-coil that is exported through the barrel (Meng et al., 2006). These features imply an intrinsic capacity for molecular permeation when the lumen is unoccupied and suggest tunability in size exclusion and electrostatic interactions with solutes. These traits are of interest for nanopore engineering. However, analyses of the central lumen of the β-barrels of TAAs have been limited because deletion of the coiled-coil in the transmembrane domain of Hia from *Haemophilus influenzae*, the first structurally characterized transmembrane domain of TAAs, prevented proper expression in bacteria (Meng et al., 2006). Recently, we determined the crystal structure of the transmembrane domain of AtaA (Yoshimoto et al., 2025), a TAA from the highly adhesive bacterium *Acinetobacter* sp. Tol 5 (Ishii et al., 2022; Yoshimoto et al., 2024; Ohara et al., 2025). To our knowledge, this represents the third structure of a TAA transmembrane β-barrel following studies on Hia and YadA from *Yersinia enterocolitica* (Meng et al., 2006; Shahid et al., 2012), and together with molecular dynamics simulations in a lipid membrane, provides an initial foundation for protein design and engineering.

Here, we removed the coiled-coil region that occupies the central lumen of the β-barrel from the transmembrane domain of AtaA to create an open β-barrel pore, termed AtaApore. The polypeptide of AtaApore was produced using a cell-free expression system, reconstituted into lipid membranes, and evaluated by electrophysiological measurements. Molecular dynamics simulations were also used to assess its ion transport properties. To our knowledge, this study represents the first electrophysiological characterization of a nanopore derived from a type Vc SS.

## 2 Materials and methods

### 2.1 Plasmid construction and antiserum generation

To construct an expression plasmid for AtaApore, a DNA fragment encoding the T7 promoter, Shine–Dalgarno (SD) sequence, N-terminal His-tag, AtaA_3575–3630_, and T7 terminator was inserted into the cloning site of the pUCFa vector. To optimize the codon usage for PURE*frex* 2.0 (GeneFrontier, Japan), the GeneFrontier DNA template design support service was utilized. The DNA sequence of the plasmid, pUCFa-AtaApore, is provided in Supplementary Note S1.

To produce a recombinant protein of the His-tagged AtaA_3524–3630_ as an antigen, an overnight culture of BL21 (DE3; pIBA-His-AtaA_3524–3630_) (Yoshimoto et al., 2025) was diluted 1:100 in LB medium and incubated for 3 h at 37 °C. After incubation, anhydrotetracycline (AHTC) (final concentration: 0.2 μg/mL) was added to the medium, and the mixture was further incubated at 28 °C for 12 h. The cells were harvested by centrifugation at 5,000 × *g* at 4 °C for 15 min, resuspended in lysis buffer (25 mM Tris-HCl, 150 mM NaCl, 20 mM imidazole, pH 9.0) supplemented with 0.1 mg/mL lysozyme, and lysed using a high-pressure homogenizer (LAB 2000; SMT Co., Tokyo, Japan) at 1,000 bar for 10 min. After centrifugation at 5,000 × *g* at 4 °C for 15 min, the supernatant was ultracentrifuged at 100,000 × *g* at 4 °C for 2 h, and the precipitate was solubilized with 5% Elugent (Merck, Darmstadt, Germany) and suspended in Elugent buffer A (25 mM Tris-HCl, 150 mM NaCl, 20 mM imidazole, 0.5% Elugent, pH 9.0). The suspension was loaded onto a Ni-NTA column (Qiagen, Venlo, Netherlands), and unbound proteins were washed off with Elugent buffer A. The bound proteins were eluted with Elugent buffer A supplemented with 300 mM imidazole and loaded onto a HiLoad 26/60 Superdex 75 pg column (Cytiva, Marlborough, MA) equilibrated with Elugent buffer B (25 mM Tris-HCl, 150 mM NaCl, 0.5% Elugent, pH 9.0). The purified protein was used to generate a rabbit polyclonal anti-transmembrane domain antiserum.

### 2.2 Cell-free protein expression

AtaApore was synthesized using a cell-free protein expression system, PURE*frex* 2.0 (GeneFrontier). A final DNA concentration of 20 ng/μL was used for expression confirmation, and 50 ng/μL was used for the liposomal permeation assay and electrophysiological measurement, respectively. The reaction mixture containing Solutions I–III (PURE*frex* 2.0) and the DNA was incubated at 37 °C for 4 h. Protein expression was evaluated by SDS–PAGE and Blue Native PAGE, followed by CBB staining and Western blotting. For SDS–PAGE, the protein sample was mixed with 2 × Tricine SDS sample buffer (0.2 M Tris-HCl, 2% SDS, 40% glycerol, 0.04% CBB G-250, 2% β-mercaptoethanol, pH 8.45) and heated at 98 °C for 5 min. Electrophoresis was conducted using a 16% polyacrylamide gel (separating gel) and a 4% polyacrylamide gel (stacking gel) under constant current conditions (20 mA per gel) with anode buffer (100 mM Tris-HCl, pH 8.9) and cathode buffer (100 mM Tris, 100 mM Tricine, 0.1% SDS). Protein bands were visualized by CBB staining. For Blue Native PAGE, the protein sample was mixed with native sample buffer (WSE-7011; ATTO, Tokyo, Japan) and incubated on ice for 5 min. The sample was loaded onto a 10%–20% gradient native gel (e-PAGEL; ATTO) in Blue Native running buffer supplemented with additive in the cathode chamber (WSE-7057; ATTO). Gels were subjected to CBB staining or Western blotting using the rabbit polyclonal anti-transmembrane domain antiserum and an anti-rabbit HRP-conjugated antibody.

### 2.3 Liposomal permeation assay

Calcein-encapsulated liposomes were prepared by the hydration-extrusion method (MacDonald et al., 1991; Berger et al., 2001). 150 μL of 50 mg/mL POPC (Avanti Polar Lipids, Alabaster, AL, United States) dissolved in chloroform was transferred to a glass vial and dried under a stream of N_2_ gas to ensure complete removal of chloroform. The resulting lipid film was hydrated with 300 μL of calcein buffer (10 mM HEPES, 50 mM Glucose, 30 mM calcein, pH 7.8) and vortexed thoroughly. The suspension was sonicated and subjected to five freeze-thaw cycles using liquid nitrogen and a 65 °C water bath. The liposomes were extruded 40 times through a 100 nm polycarbonate filter membrane using a Mini-Extruder (Avanti Polar Lipids). After a centrifugation at 15,000 × *g* for 20 min at 4 °C, the supernatant containing liposomes was transferred to a new tube and mixed with 1.7 mL of wash buffer (10 mM HEPES, 188 mM NaCl, pH 7.8). The suspension was ultracentrifuged at 180,000 × *g* for 40 min at 4 °C. The supernatant was discarded, and the liposome pellet was resuspended in 2 mL of wash buffer. This washing step was repeated three times in total. The final pellet was resuspended in 300 μL of wash buffer to yield a liposome suspension with an approximate lipid concentration of 10 mg/mL.

The liposomal permeation assay was performed using an *in vitro* protein synthesis system. 9 μL of the calcein-encapsulated liposomes were mixed with PURE*frex* 2.0 Solutions I–III for a total reaction volume of 30.5 µL. Plasmid DNA encoding AtaApore was added to initiate protein expression. Reactions were incubated at 37 °C for 4 h. After incubation, 5 µL of the reaction mixture was diluted with 2 mL of wash buffer and ultracentrifuged at 180,000 × *g* for 70 min at 4 °C. The fluorescence intensity of the resulting supernatant was measured using a microplate reader (excitation = 500 nm, emission = 534 nm, gain = 68; Infinite 200PRO M Plex, Tecan, Mannedorf, Switzerland).

### 2.4 Electrophysiological measurement

Electrophysiological measurements were performed using a planar lipid bilayer device as previously described (Kawano et al., 2013; Kawano et al., 2014). The microdevice consisted of a 6.0 mm-thick polymethylmethacrylate (PMMA) plate (10 × 10 mm) with two 2.0 mm-diameter, 4.5 mm-deep chambers separated by a PMMA partition, each with a perforation for Ag/AgCl electrodes. A parylene-C polymer film (5 µm thick) containing a single 100 µm-diameter pore was fabricated by photolithography and assembled between two 0.2 mm-thick PMMA sheets to complete the device.

Proteoliposomes containing AtaApore were prepared by expressing AtaApore in the presence of liposomes. In brief, 1,2-dioleoyl-*sn*-glycero-3-phosphoethanolamine (DOPE; Avanti Polar Lipids), 1,2-dioleoyl-*sn*-glycero-3-phosphatidylserine (DOPS; Funakoshi Co., Ltd., Tokyo, Japan), 1,2-dioleoyl-*sn*-glycero-3-phosphocholine (DOPC; Avanti Polar Lipids), and cholesterol (Olbracht Serdary Research Laboratories, Toronto, ON, Canada) were dissolved in chloroform at 50 mg/mL and mixed at a ratio of 5:2:1:1 (Kawano et al., 2013) and transferred to a glass vial and dried under a stream of N_2_ gas. The resulting lipid film was hydrated with 300 μL of HEPES buffer (10 mM HEPES, 50 mM glucose, pH 7.4) and subjected to five freeze-thaw cycles using liquid nitrogen and a 65 °C water bath. The lipid suspension was then extruded 40 times through a 100 nm polycarbonate filter membrane using a Mini-Extruder (Avanti Polar Lipids). The liposome suspension was ultracentrifuged at 180,000 × *g* for 30 min, and the pellet was resuspended in 300 µL of HEPES buffer. Ten microliters of the liposome was added to the reaction mixture of PURE*frex* 2.0 supplemented with 50 ng/μL pUCFa-AtaApore and incubated at 37 °C for 4 h. The prepared proteoliposomes were then fused with planar lipid bilayers composed of DOPC. The channel current was monitored using a JET patch-clamp amplifier (Tecella, Foothill Ranch, CA, United States). Signals were lowpass filtered at 4 kHz and sampled at 20 kHz. A constant voltage of +150 mV was applied to the recording chamber, while the ground chamber was maintained at 0 mV. Both chambers contained a recording buffer composed of 10 mM HEPES-KOH (pH 7.4) and 1 M KCl. Data were analyzed using pCLAMP ver 10.7 (Molecular Devices, San Jose, CA, United States). All measurements were performed at 22 °C ± 2 °C.

### 2.5 Structural modeling

The trimeric structure of AtaApore was predicted using AlphaFold2 (multimer mode) (Evans et al., 2022). The input sequence corresponded to residues 3575–3630 of AtaA, as described in Supplementary Note S2. The predicted trimer model was embedded into a lipid bilayer using CHARMM-GUI Membrane Builder (Wu et al., 2014), in which both the upper and lower leaflets were composed of 100% POPC. The systems were parameterized with the CHARMM36 m force field (Huang et al., 2017) and the CHARMM-modified TIP3P water model (Jorgensen et al., 1983). Water molecules and potassium chloride (KCl) ions were added to neutralize the system charge, and the final ion concentration was set to 1.0 M.

The R3597G/R3622G mutant model was generated using CHARMM-GUI (Jo et al., 2008) based on the wild-type structure. This system was embedded in a 100% POPC bilayer and solvated in 1.0 M KCl, following the same protocol as for the wild type.

### 2.6 MD simulations

All MD simulations were performed using GROMACS 2020 (Abraham et al., 2015). The temperature was maintained at 298.15 K using the V-rescale thermostat (Bussi et al., 2007), and the pressure was controlled at 1.0 bar using the Parrinello–Rahman barostat when applicable (Parrinello and Rahman, 1981). All bonds involving hydrogen atoms were constrained using the LINCS algorithm (Hess et al., 1997). Electrostatic interactions were treated with the particle mesh Ewald (PME) method (Darden et al., 1993), and both Lennard–Jones and real-space electrostatic interactions were truncated at a 1.2 nm cutoff. The Fourier grid spacing for reciprocal-space PME calculations was set to 0.1 nm, and all simulations were carried out with a time step of 2 fs.

To assess the structural stability of AtaApore embedded in a POPC lipid bilayer, an MD simulation was performed for 1,000 ns, and the root mean square deviation (RMSD) of protein atoms was analyzed. All trajectories were visualized using VMD (Humphrey et al., 1996).

To examine ion transport properties, simulations were conducted under a +150 mV transmembrane potential using an NVT ensemble. A constant electric field was applied along the Z-axis, corresponding to the potential difference between the top and bottom of the simulation box. Heavy atoms in the protein backbone (N, Cα, C, and O) were position-restrained with a force constant of 1,000 kJ mol^-1^ nm^-2^ to maintain the overall pore geometry, and each system was simulated for 500 ns in triplicate with independent initial velocities.

The ionic current (*I*) and conductance (*G*) were obtained from the number of K^+^ and Cl^−^ ions traversing the pore along the Z-axis over the simulation period (Δ*t*), according to:

$$I=\frac{\left(N_{K}+N_{Cl}\right)\cdot e}{\Delta t}$$

where *e* is the elementary charge (1.602 × 10^−19^ C), *N*_*K*_ is the count of K^+^ ions passing in the +Z direction, and *N*_*Cl*_ is the count of Cl^−^ ions passing in the -Z direction. The conductance (*G*) was then calculated using Ohm’s law:

$$G=\frac{I}{V}$$

where *V* is the applied voltage (+150 mV). The ionic currents and conductance values were averaged over three independent simulations, and the results were reported as mean ± standard deviation.

### 2.7 Bioinformatics

Amino acid sequences of the transmembrane domain of well-characterized TAAs were collected manually from the UniProt Knowledgebase (The UniProt Consortium, 2025). The multiple sequence alignment was generated using Clustal Omega (Sievers et al., 2011) on the MPI Bioinformatics Toolkit (Zimmermann et al., 2018) and visualized by Jalview (Waterhouse et al., 2009).

### 2.8 Statistical analysis

All quantitative data are presented as mean ± SEM unless otherwise stated. For electrophysiological measurements, event duration times were compared between AtaApore and control experiments using the Brunner–Munzel test (Brunner and Munzel, 2000). Conductance values were summarized as median and interquartile range (IQR) due to non-normal distributions. For liposome permeation assays, statistical significance was evaluated using Student’s *t*-test. A *p*-value <0.05 was considered statistically significant.

## 3 Results and discussion

### 3.1 Cell-free expression of AtaApore

**FIGURE 1:**
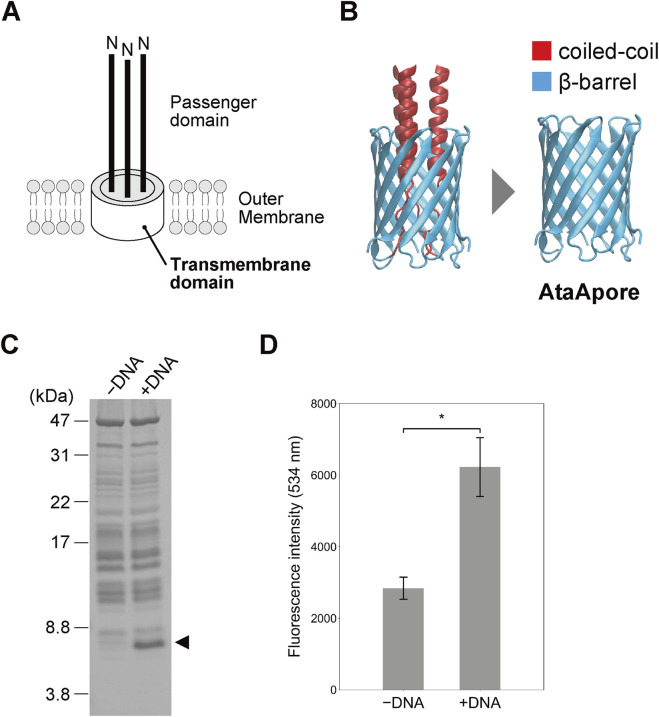
Expression and membrane reconstitution of AtaApore. **(A)** Schematic representation of the C-terminal region of AtaA. **(B)** Design of the β-barrel pore, AtaApore, by deleting the lumen-occupying coiled-coil segment that traverses the transmembrane β-barrel of AtaA. **(C)** Cell-free expression of AtaApore. Total proteins from reactions lacking (−) or containing (+) the DNA encoding AtaApore were analyzed by SDS–PAGE followed by CBB staining. The arrowhead indicates the bands expected to correspond to monomeric AtaApore. **(D)** Liposome permeation assay. Fluorescence intensity of the supernatant from reactions lacking (−) or containing (+) the DNA encoding AtaApore was measured after removing liposomes encapsulating calcein. Data are expressed as mean ± SEM (n = 3). Statistical significance, *p < 0.05, Student’s *t*-test.

We designed a β-barrel nanopore, AtaApore, by deleting the internal coiled-coil that occludes the pore lumen (Figures 1A,B). We initially attempted to express AtaApore in *E. coli*, but no protein production was detected. Therefore, we turned to a cell-free expression system. The gene encoding AtaApore was codon-optimized, placed under a T7 promoter, and cloned into the pUCFa plasmid (Supplementary Note S1). After cell-free expression with the DNA encoding AtaApore, the resulting protein samples were analyzed by SDS–PAGE followed by Coomassie Brilliant Blue (CBB) staining. A distinct band at the expected apparent molecular weight of monomeric AtaApore (6.5 kDa) was detected only in the reaction with the DNA, indicating its successful expression (Figure 1C). Blue Native PAGE followed by Western blotting further confirmed the expression of AtaApore and indicated the formation of a trimeric complex (Supplementary Figure S1).

Next, AtaApore was expressed in the presence of liposomes, and its pore-forming ability was evaluated using a liposome permeation assay. In this assay, pore formation in the lipid membrane of liposomes encapsulating a fluorescent dye led to leakage of the dye, resulting in increased fluorescence of the supernatant of the solution. After the expression of AtaApore using the cell-free expression system, the supernatant of the reaction showed higher fluorescence intensity than the control reaction lacking the plasmid encoding AtaApore (Figure 1D). These results indicate that AtaApore has the ability to form a pore in the lipid membrane.

### 3.2 Electrophysiological characterization

**FIGURE 2:**
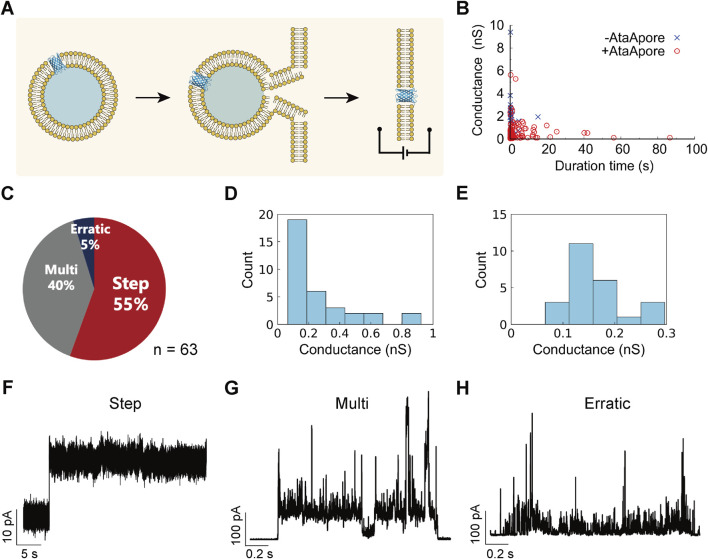
Electrophysiological measurements of AtaApore. **(A)** Schematic illustration of proteoliposome fusion with a planar lipid bilayer for AtaApore delivery. **(B)** Mean open-state conductance and duration times extracted from each current trace. **(C)** Classification of the signals into three categories based on stability and level multiplicity. **(D)** Histogram of conductance values obtained from step-like current signals of AtaApore (n = 34). **(E)** Expanded conductance histogram in the range of 0–0.3 nS (n = 24). **(F–H)** Representative current traces for each signal type: **(F)** stable step-like, **(G)** unstable multi-level, and **(H)** unstable erratic.

Subsequently, the electrophysiological characteristics of AtaApore were assessed using a planar lipid bilayer system (Figure 2A) (Kawano et al., 2013; Kawano et al., 2014). For the measurements, AtaApore was first expressed with liposomes, yielding proteoliposomes that enabled pore formation and membrane insertion prior to fusion with the planar lipid bilayer. The proteoliposomes containing AtaApore were fused with planar lipid bilayers composed of 1,2-dioleoyl-*sn*-glycero-3-phosphocholine (DOPC). Signals consistent with stable single-channel activity were detected under a voltage of +150 mV. To verify that the observed signals were due to AtaApore and not transient liposome-fusion events, we analyzed duration times and open-state conductance calculated from current values (Figure 2B). In control experiments without AtaApore, more than 80% of events lasted less than 1 s, indicating transient liposome fusion. In contrast, 63 of 116 events with AtaApore persisted for more than 1 s and showed a longer lifetime than the control (*p* < 0.01), indicating pore formation in the lipid membrane. These events were classified into three patterns as described previously (Shimizu et al., 2022): step-like (a stable open state with a single conductance level), multi-level (two or more conductance states within a single opening), and erratic (unstable, flickering openings) (Figure 2C). Step-like events accounted for approximately 55% of total events. This ratio is comparable to those reported for other biological nanopores such as FraC (62%), PFN (60%), and SLO (71%), as characterized in previous studies (Watanabe et al., 2017), indicating that AtaApore exhibits a similar level of pore stability to established biological pores. From the step-like events, the single-channel conductance of AtaApore was calculated as median (interquartile range, IQR) = 0.17 (0.12–0.32) nS (Figures 2D,E), demonstrating that the engineered β-barrel can form stable nanopores with measurable conductance. Representative traces are shown in Figure 2F (step-like), Figure 2G (multi-level), and Figure 2H (erratic). These multiple or erratic signals may have resulted from monomeric AtaApore or from unstable multimers. The slightly broad conductance distribution observed even for step-like events (Figures 2D,E) may also, at least in part, reflect the occasional formation of larger multimeric assemblies or the simultaneous insertion of multiple pores.

The conductance of AtaApore (0.17 nS) is lower than those of established nanopores such as MspA (∼4.9 nS) (Butler et al., 2008), α-hemolysin (∼1.0 nS) (Akeson et al., 1999), and the synthetic β-barrel SVG28 (∼1.0 nS) (Shimizu et al., 2022), but comparable to that of bacterial outer membrane protein A (OmpA, 0.26–0.32 nS) (Arora et al., 2000). Functional nanopores have also been reported from type Va secretion systems, including NalP and Hbp, whose β-barrel domains form membrane pores upon removal of their passenger domains (Oomen et al., 2004; Roussel-Jazédé et al., 2011). In these systems, the conductance remains low (∼0.2 nS) when the internal α-helix is retained within the barrel, but increases to ∼1.3 nS when the α-helix is removed. In contrast, even after removal of the internal coiled-coil, AtaApore still exhibited a relatively low conductance. This difference may reflect intrinsic structural features of β-barrels of type Vc SS, such as the unique trimeric arrangement of subunits. From a sensing perspective, the low conductance of AtaApore reflects its narrow lumen and may be advantageous for high sensitivity nanopore detection. Smaller pore diameters enhance current blockade amplitudes and signal-to-noise ratios, facilitating small-molecule sensing, particularly for analytes such as ATP, amino acids, nucleotides, and small drug molecules, as described in previous studies (Su et al., 2019; Chingarande et al., 2023; Wang et al., 2024). However, because the open-pore current is relatively low, analyte permeation or event frequency could become limiting factors, and these aspects should be optimized depending on the intended analyte and measurement goals.

### 3.3 MD simulations of AtaApore

**FIGURE 3:**
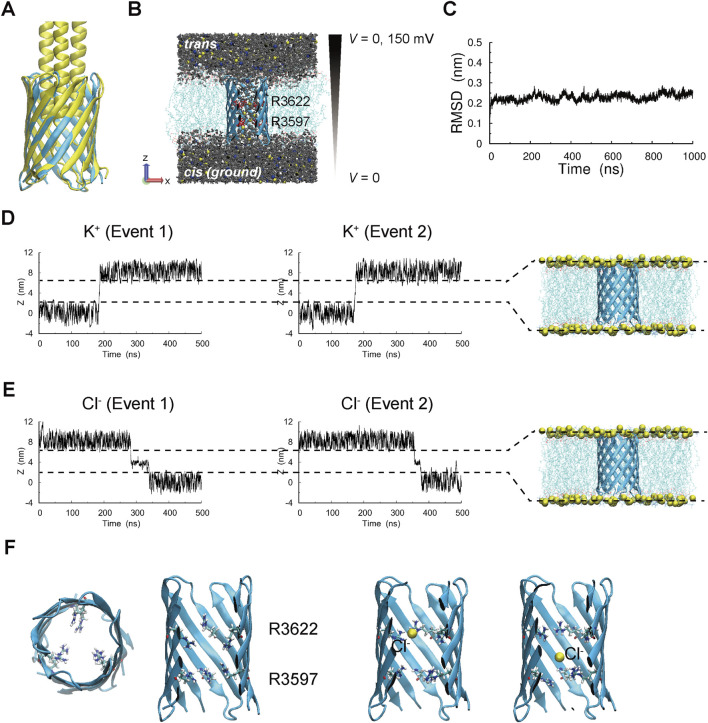
MD simulations of ion transport through AtaApore. **(A)** Comparison of the predicted structure of AtaApore (cyan) with the crystal structure of the transmembrane domain of AtaA (9VNJ, yellow). **(B)** Simulation system of AtaApore embedded in a POPC bilayer, used for both equilibrium (0 mV) and field-applied (+150 mV) simulations. When a transmembrane potential was applied, an electric field was oriented along the -Z direction, corresponding to a potential difference of +150 mV between the *trans* (top) and *cis* (bottom, ground) sides. Water molecules, K^+^, and Cl^−^ are shown as gray, blue, and yellow spheres, respectively. Residues R3597 and R3622 are represented in the Licorice style in VMD. **(C)** Structural stability of AtaApore evaluated by RMSD over a 1,000 ns MD simulation. **(D,E)** Representative plots showing positional changes of K^+^ and Cl^−^ ions along the Z-axis during a 500 ns simulation under a +150 mV transmembrane potential. Two independent trajectories are shown for each ion species. The Z-axis is perpendicular to the membrane plane. **(F)** Structural representation of the constriction region formed by R3597 and R3622 residues within AtaApore, with snapshots showing a Cl^−^ ion transiently trapped at the constriction.

The trimeric structure of AtaApore was predicted using AlphaFold2 (Figure 3A, cyan). This model, which consists of a 12-stranded β-barrel formed by four strands contributed by each of the three subunits surrounding a central lumen, closely matches the overall architecture of the crystal structure of the AtaA transmembrane domain (Figure 3A, yellow) (Yoshimoto et al., 2025). The relative orientation of the subunits, the barrel diameter, and the interfacial β-sheet arrangement were nearly identical between the two structures, suggesting that the removal of the internal coiled-coil does not alter the overall trimeric β-barrel architecture. To examine the stability of this model, we performed a 1,000 ns molecular dynamics (MD) simulation in which AtaApore was embedded in a 1-palmitoyl-2-oleoyl-phosphatidylcholine (POPC) lipid bilayer, which is widely used in nanopore simulations due to its biological relevance and well-characterized physicochemical properties (Figure 3B). Throughout the simulation, the root mean square deviation (RMSD) of AtaApore remained low and converged (Figure 3C), and the three subunits stayed tightly associated without dissociation or deformation of the barrel, suggesting that the transmembrane domain of AtaA remains stable even after the removal of the lumen-occluding coiled-coil.

To investigate the ion-transport properties of AtaApore, we performed three independent 500 ns MD simulations with 1 M KCl under an applied transmembrane potential of +150 mV. Both K^+^ and Cl^−^ ions successfully translocated through the pore, with K^+^ ions selectively moving from the *cis* side at 0 mV to the *trans* side at +150 mV and Cl^−^ in the reverse direction (Figures 3B,D,E; Supplementary Figure S2, Supplementary Movie S1). The number of K^+^ and Cl^−^ ions traversing the pore along the Z-axis was counted to estimate ionic current. The calculated average current was 41.54 ± 2.08 pA, corresponding to a conductance of 0.28 ± 0.014 nS. While the simulated conductance was higher than the measured value, similar overestimations have often been observed in nanopore simulations due to enhanced electroosmotic flow and force field-dependent ion mobility (Pothula et al., 2016; Prajapati et al., 2022). Positional restraints applied to the Cα atoms of AtaApore may also have facilitated ion passage. Next, we focused on the behavior of ions during translocation. Trajectories of Cl^−^ ions exhibited frequent transient trapping events within the pore (Figure 3E). These events consistently occurred in the region between R3597 and R3622 within the inner lumen of the β-barrel, slowing translocation and prolonging residence times at this site (Figure 3F, Supplementary Movie S2). In contrast, such trapping was not observed for K^+^ ions (Figure 3D; Supplementary Figure S2). Given that charged constrictions regulate transport and molecular discrimination in other biological nanopores (Van der Verren et al., 2020; Zhou et al., 2020), these observations suggest that R3597 and R3622 may contribute to electrostatic interactions and influence ion selectivity in AtaApore.

**FIGURE 4:**
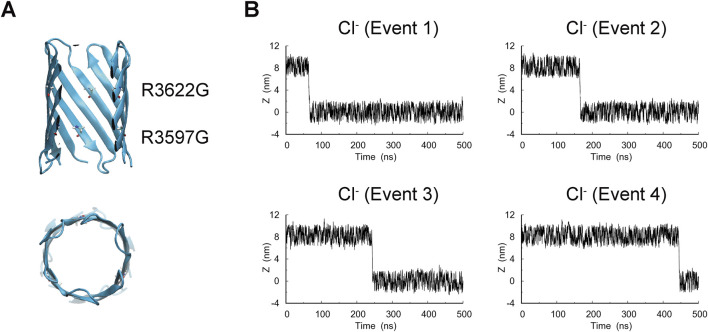
MD simulation of the R3597G/R3622G mutant. **(A)** Model structure of the R3597G/R3622G mutant of AtaApore. The mutated residues are represented in the Licorice style in VMD. **(B)** Representative plots showing positional changes of Cl^−^ ions along the Z-axis during a 500 ns simulation under a +150 mV transmembrane potential.

To examine whether R3597 and R3622 are responsible for the observed anion trapping and low conductance, we constructed an R3597G/R3622G double-mutant model and performed MD simulations using the same protocol to assess ion conduction (Figure 4A). We observed frequent translocation events of K^+^ and Cl^−^ ions, and the calculated current was 60.02 ± 0.67 pA, corresponding to a conductance of 0.40 ± 0.004 nS, which was higher than that of the wild-type pore. The Cl^−^ trapping observed in the wild-type pore was not observed in the mutant (Figure 4B), indicating that R3597 and R3622 influence ion passage dynamics and conductance. Such narrow and structurally defined nanopores are increasingly exploited for high-resolution sensing of small molecules, peptides, and protein conformations (Ratinho et al., 2025), and AtaApore may provide a new scaffold for these applications. Sequence alignment of TAA transmembrane domains further shows that these arginine residues are conserved in *Acinetobacter*, which could favor transient analyte retention beneficial for nanopore sensing, although the biological implications remain to be determined (Supplementary Figure S3).

In addition to its potential use in single-molecule nanopore sensing, AtaApore may also be applied as a transmembrane transporter in liposomal or synthetic cell systems. Because it can insert into lipid bilayers and permit passive ion transport, AtaApore could, in principle, be used to modulate ion gradients or enable limited molecular exchange across vesicle membranes, thereby expanding the set of channels available for synthetic biology.

Beyond such application-oriented uses, the open β-barrel pore may also serve as a practical model for probing the type Vc SS, which is not as well characterized as the type Va SS. TAAs are thought to secrete their passenger domains through the β-barrel by a hairpin mechanism (Mikula et al., 2012; Chauhan et al., 2019), but the underlying dynamics remain unclear. The absence of the lumen-occluding coiled-coil in AtaApore could permit peptide translocation while monitoring ionic currents in real time. Corresponding changes in current amplitude and duration time could reflect transient interactions within the β-barrel. Such measurements may enable single-molecule analyses of autotransport dynamics, as has been studied for type Va SS (Kang’ethe and Bernstein, 2013; Drobnak et al., 2015), and could complement genetic and structural studies to uncover the mechanisms of type Vc SS.

## 4 Conclusion

In this work, we designed AtaApore, the β-barrel of a trimeric autotransporter adhesin that lacks an internal coiled-coil passenger domain. By combining cell-free protein expression, membrane reconstitution, electrophysiological measurements, and molecular dynamics simulations, we showed that the designed β-barrel pore is capable of ion conduction. Its low conductance and defined constriction site will offer opportunities for rational tuning and high-resolution sensing. These features make AtaApore a promising scaffold for high-resolution sensing of small molecules, peptides, and transient conformational states. To our knowledge, this is the first characterization of a nanopore derived from a type Vc SS, offering a simplified model to explore the mechanism of type Vc secretion and expanding the available repertoire of nanopore scaffolds for biosensing and bottom-up synthetic biology.

## Data Availability

The original contributions presented in the study are included in the article/Supplementary Material, further inquiries can be directed to the corresponding author.

## References

[B1] Abraham M. J. Murtola T. Schulz R. Páll S. Smith J. C. Hess B. (2015). GROMACS: high performance molecular simulations through multi-level parallelism from laptops to supercomputers. SoftwareX 1–2, 19–25. 10.1016/J.SOFTX.2015.06.001 1

[B2] Akeson M. Branton D. Kasianowicz J. J. Brandin E. Deamer D. W. (1999). Microsecond time-scale discrimination among polycytidylic acid, polyadenylic acid, and polyuridylic acid as homopolymers or as segments within single RNA molecules. Biophys. J. 77 (6), 3227–3233. 10.1016/S0006-3495(99)77153-5 10585944 PMC1300593

[B3] Arora A. Rinehart D. Szabo G. Tamm L. K. (2000). Refolded outer membrane protein A of *Escherichia coli* forms ion channels with two conductance states in planar lipid bilayers. J. Biol. Chem. 275 (3), 1594–1600. 10.1074/jbc.275.3.1594 10636850

[B4] Bentancor L. V. Camacho-Peiro A. Bozkurt-Guzel C. Pier G. B. Maira-Litrán T. (2012). Identification of ata, a multifunctional trimeric autotransporter of *Acinetobacter baumannii* . J. Bacteriol. 194 (15), 3950–3960. 10.1128/jb.06769-11 22609912 PMC3416510

[B5] Berger N. Sachse A. Bender J. Schubert R. Brandl M. (2001). Filter extrusion of liposomes using different devices: comparison of liposome size, encapsulation efficiency, and process characteristics. Int. J. Pharm. 223 (1), 55–68. 10.1016/s0378-5173(01)00721-9 11451632

[B6] Brunner E. Munzel U. (2000). The nonparametric behrens-fisher problem: asymptotic theory and a small-sample approximation. Biom. J. 42 (1), 17–25. 10.1002/(sici)1521-4036(200001)42:1<17::aid-bimj17>3.0.co;2-u

[B7] Bussi G. Donadio D. Parrinello M. (2007). Canonical sampling through velocity rescaling. J. Chem. Phys. 126 (1), 014101. 10.1063/1.2408420 17212484

[B8] Butler T. Z. Pavlenok M. Derrington I. M. Niederweis M. Gundlach J. H. (2008). Single-molecule DNA detection with an engineered MspA protein nanopore. Proc. Natl. Acad. Sci. U.S.A. 105 (52)**,** 20647–20652. 10.1073/pnas.0807514106 19098105 PMC2634888

[B9] Cao C. Cirauqui N. Marcaida M. J. Buglakova E. Duperrex A. Radenovic A. (2019). Single-molecule sensing of peptides and nucleic acids by engineered aerolysin nanopores. Nat. Commun. 10 (1), 4918. 10.1038/s41467-019-12690-9 31664022 PMC6820719

[B10] Chauhan N. Hatlem D. Orwick-Rydmark M. Schneider K. Floetenmeyer M. van Rossum B. (2019). Insights into the autotransport process of a trimeric autotransporter, yersinia adhesin A (YadA). Mol. Microbiol. 111 (3), 844–862. 10.1111/mmi.14195 30600549

[B11] Chingarande R. G. Tian K. Kuang Y. Sarangee A. Hou C. Ma E. (2023). Real-time label-free detection of dynamic aptamer–small molecule interactions using a nanopore nucleic acid conformational sensor. Proc. Natl. Acad. Sci. U.S.A. 120 (24), e2108118120. 10.1073/pnas.2108118120 37276386 PMC10268594

[B12] Costa T. R. D. Felisberto-Rodrigues C. Meir A. Prevost M. S. Redzej A. Trokter M. (2015). Secretion systems in Gram-negative bacteria: structural and mechanistic insights. Nat. Rev. Microbiol. 13 (6), 343–359. 10.1038/nrmicro3456 25978706

[B13] Darden T. York D. Pedersen L. (1993). Particle mesh ewald: an *N*⋅log(*N*) method for Ewald sums in large systems. J. Chem. Phys. 98 (12), 10089–10092. 10.1063/1.464397

[B14] Drobnak I. Braselmann E. Clark P. L. (2015). Multiple driving forces required for efficient secretion of autotransporter virulence proteins. J. Biol. Chem. 290 (16), 10104–10116. 10.1074/jbc.M114.629170 25670852 PMC4400326

[B15] Duret G. Szymanski M. Choi K.-J. Yeo H.-J. Delcour A. H. (2008). The TpsB translocator HMW1B of *Haemophilus influenzae* forms a large conductance channel. J. Biol. Chem. 283 (23), 15771–15778. 10.1074/jbc.M708970200 18403374 PMC2414297

[B16] Evans R. O’Neill M. Pritzel A. Antropova N. Senior A. Green T. (2022). Protein complex prediction with AlphaFold-Multimer. bioRxiv, 463034. 10.1101/2021.10.04.463034

[B17] Fan E. Fiedler S. Jacob-Dubuisson F. Müller M. (2012). Two-partner secretion of gram-negative bacteria: a single β-barrel protein enables transport across the outer membrane. J. Biol. Chem. 287 (4), 2591–2599. 10.1074/jbc.M111.293068 22134917 PMC3268418

[B18] Goyal P. Krasteva P. V. Van Gerven N. Gubellini F. Van den Broeck I. Troupiotis-Tsaïlaki A. (2014). Structural and mechanistic insights into the bacterial amyloid secretion channel CsgG. Nature 516 (7530), 250–253. 10.1038/nature13768 25219853 PMC4268158

[B19] Green E. R. Mecsas J. (2016). Bacterial secretion systems: an overview. Microbiol. Spectr. 4 (1), vmbf-0012–2015. 10.1128/microbiolspec.VMBF-0012-2015 26999395 PMC4804464

[B20] Hess B. Bekker H. Berendsen H. J. Fraaije J. G. (1997). LINCS: a linear constraint solver for molecular simulations. J. Comput. Chem. 18 (12), 1463–1472. 10.1002/(SICI)1096-987X(199709)18:12<1463::AID-JCC4>3.0.CO;2-H

[B21] Huang J. Rauscher S. Nawrocki G. Ran T. Feig M. de Groot B. L. (2017). CHARMM36m: an improved force field for folded and intrinsically disordered proteins. Nat. Methods 14 (1), 71–73. 10.1038/nmeth.4067 27819658 PMC5199616

[B22] Humphrey W. Dalke A. Schulten K. (1996). VMD: visual molecular dynamics. J. Mol. Graph. 14 (1), 33–38. 10.1016/0263-7855(96)00018-5 8744570

[B23] Ishii S. Yoshimoto S. Hori K. (2022). Single-cell adhesion force mapping of a highly sticky bacterium in liquid. J. Colloid Interface Sci. 606, 628–634. 10.1016/j.jcis.2021.08.039 34416455

[B24] Jo S. Kim T. Iyer V. G. Im W. (2008). CHARMM-GUI: a web-based graphical user interface for CHARMM. J. Comput. Chem. 29 (11), 1859–1865. 10.1002/jcc.20945 18351591

[B25] Jorgensen W. L. Chandrasekhar J. Madura J. D. Impey R. W. Klein M. L. (1983). Comparison of simple potential functions for simulating liquid water. J. Chem. Phys. 79 (2), 926–935. 10.1063/1.445869

[B26] Kang'ethe W. Bernstein H. D. (2013). Stepwise folding of an autotransporter passenger domain is not essential for its secretion. J. Biol. Chem. 288 (49), 35028–35038. 10.1074/jbc.M113.515635 24165126 PMC3853255

[B27] Kawano R. Tsuji Y. Sato K. Osaki T. Kamiya K. Hirano M. (2013). Automated parallel recordings of topologically identified single ion channels. Sci. Rep. 3 (1), 1995. 10.1038/srep01995 23771282 PMC3683667

[B28] Kawano R. Tsuji Y. Kamiya K. Kodama T. Osaki T. Miki N. (2014). A portable lipid bilayer system for environmental sensing with a transmembrane protein. PLoS One 9 (7), e102427. 10.1371/journal.pone.0102427 25072468 PMC4114450

[B29] Leo J. C. Grin I. Linke D. (2012). Type V secretion: mechanism(s) of autotransport through the bacterial outer membrane. Philos. Trans. R. Soc. Lond. B Biol. Sci. 367 (1592), 1088–1101. 10.1098/rstb.2011.0208 22411980 PMC3297439

[B30] Leyton D. L. Rossiter A. E. Henderson I. R. (2012). From self sufficiency to dependence: mechanisms and factors important for autotransporter biogenesis. Nat. Rev. Microbiol. 10 (3), 213–225. 10.1038/nrmicro2733 22337167

[B31] Lin J. Huang S. Zhang Q. (2002). Outer membrane proteins: key players for bacterial adaptation in host niches. Microbes Infect. 4 (3), 325–331. 10.1016/s1286-4579(02)01545-9 11909743

[B32] Linke D. Riess T. Autenrieth I. B. Lupas A. Kempf V. A. (2006). Trimeric autotransporter adhesins: variable structure, common function. Trends Microbiol. 14 (6), 264–270. 10.1016/j.tim.2006.04.005 16678419

[B33] Lu C. Bonini A. Viel J. H. Maglia G. (2025). Toward single-molecule protein sequencing using nanopores. Nat. Biotechnol. 43 (3), 312–322. 10.1038/s41587-025-02587-y 40097683 PMC12006967

[B34] MacDonald R. C. MacDonald R. I. Menco B. P. M. Takeshita K. Subbarao N. K. Hu L.-r. (1991). Small-volume extrusion apparatus for preparation of large, unilamellar vesicles. Biochim. Biophys. Acta Biomembr. 1061 (2), 297–303. 10.1016/0005-2736(91)90295-j 1998698

[B35] Méli A. C. Hodak H. Clantin B. Locht C. Molle G. Jacob-Dubuisson F. (2006). Channel properties of TpsB transporter FhaC point to two functional domains with a C-terminal protein-conducting pore. J. Biol. Chem. 281 (1), 158–166. 10.1074/jbc.M508524200 16284399

[B36] Meng G. Surana N. K. St Geme III. J. W. Waksman G. (2006). Structure of the outer membrane translocator domain of the *Haemophilus influenzae* Hia trimeric autotransporter. EMBO J. 25 (11), 2297–2304. 10.1038/sj.emboj.7601132 16688217 PMC1478200

[B37] Mikula K. M. Leo J. C. Łyskowski A. Kedracka-Krok S. Pirog A. Goldman A. (2012). The translocation domain in trimeric autotransporter adhesins is necessary and sufficient for trimerization and autotransportation. J. Bacteriol. 194 (4), 827–838. 10.1128/jb.05322-11 22155776 PMC3272944

[B38] Miyagi M. Yamaji M. Kurokawa N. Yohda M. Kawano R. (2025). Redesign of translocon EXP2 nanopore for detecting peptide fragments. Small Methods 9 (4), 2401562. 10.1002/smtd.202401562 39905884 PMC12020339

[B39] Motone K. Kontogiorgos-Heintz D. Wee J. Kurihara K. Yang S. Roote G. (2024). Multi-pass, single-molecule nanopore reading of long protein strands. Nature 633 (8030), 662–669. 10.1038/s41586-024-07935-7 39261738 PMC11410661

[B40] Ohara Y. Yoshimoto S. Hori K. (2025). Grid partitioning image analysis of highly aggregative bacterium *Acinetobacter* sp. Tol 5. Front. Microbiol. 16, 1637462. 10.3389/fmicb.2025.1637462 41001058 PMC12459662

[B41] Oomen C. J. van Ulsen P. Van Gelder P. Feijen M. Tommassen J. Gros P. (2004). Structure of the translocator domain of a bacterial autotransporter. EMBO J. 23 (6), 1257–1266. 10.1038/sj.emboj.7600148 15014442 PMC381419

[B42] Parrinello M. Rahman A. (1981). Polymorphic transitions in single crystals: a new molecular dynamics method. J. Appl. Phys. 52 (12), 7182–7190. 10.1063/1.328693

[B43] Piguet F. Ouldali H. Pastoriza-Gallego M. Manivet P. Pelta J. Oukhaled A. (2018). Identification of single amino acid differences in uniformly charged homopolymeric peptides with aerolysin nanopore. Nat. Commun. 9 (1), 966. 10.1038/s41467-018-03418-2 29511176 PMC5840376

[B44] Pothula K. R. Solano C. J. F. Kleinekathöfer U. (2016). Simulations of outer membrane channels and their permeability. Biochim. Biophys. Acta Biomembr. 1858 (7), 1760–1771. 10.1016/j.bbamem.2015.12.020 26721326

[B45] Prajapati J. D. Pangeni S. Aksoyoglu M. A. Winterhalter M. Kleinekathöfer U. (2022). Changes in salt concentration modify the translocation of neutral molecules through a ΔCymA nanopore in a non-monotonic manner. ACS Nano 16 (5), 7701–7712. 10.1021/acsnano.1c11471 35435659

[B46] Ratinho L. Meyer N. Greive S. Cressiot B. Pelta J. (2025). Nanopore sensing of protein and peptide conformation for point-of-care applications. Nat. Commun. 16 (1), 3211. 10.1038/s41467-025-58509-8 40180898 PMC11968944

[B47] Rollauer S. E. Sooreshjani M. A. Noinaj N. Buchanan S. K. (2015). Outer membrane protein biogenesis in Gram-negative bacteria. Philos. Trans. R. Soc. Lond. B Biol. Sci. 370 (1679), 20150023. 10.1098/rstb.2015.0023 26370935 PMC4632599

[B48] Roussel-Jazédé V. Gelder P. V. Sijbrandi R. Rutten L. Otto B. R. Luirink J. (2011). Channel properties of the translocator domain of the autotransporter Hbp of *Escherichia coli* . Mol. Membr. Biol. 28 (3), 158–170. 10.3109/09687688.2010.550328 21314477

[B49] Shahid S. A. Bardiaux B. Franks W. T. Krabben L. Habeck M. van Rossum B.-J. (2012). Membrane-protein structure determination by solid-state NMR spectroscopy of microcrystals. Nat. Methods 9 (12), 1212–1217. 10.1038/nmeth.2248 23142870

[B50] Shimizu K. Mijiddorj B. Usami M. Mizoguchi I. Yoshida S. Akayama S. (2022). *De novo* design of a nanopore for single-molecule detection that incorporates a β-hairpin peptide. Nat. Nanotechnol. 17 (1), 67–75. 10.1038/s41565-021-01008-w 34811552 PMC8770118

[B51] Sievers F. Wilm A. Dineen D. Gibson T. J. Karplus K. Li W. (2011). Fast, scalable generation of high-quality protein multiple sequence alignments using Clustal Omega. Mol. Syst. Biol. 7 (1), 539. 10.1038/msb.2011.75 21988835 PMC3261699

[B52] Su Z. Wei Y. Kang X.-f. (2019). Simultaneous high-resolution detection of bioenergetic molecules using biomimetic-receptor nanopore. Anal. Chem. 91 (23), 15255–15259. 10.1021/acs.analchem.9b04268 31665602

[B53] The UniProt Consortium (2025). UniProt: the universal protein knowledgebase in 2025. Nucleic Acids Res. 53 (D1), D609–D617. 10.1093/nar/gkae1010 39552041 PMC11701636

[B54] Van der Verren S. E. Van Gerven N. Jonckheere W. Hambley R. Singh P. Kilgour J. (2020). A dual-constriction biological nanopore resolves homonucleotide sequences with high fidelity. Nat. Biotechnol. 38 (12), 1415–1420. 10.1038/s41587-020-0570-8 32632300 PMC7610451

[B55] Veiga E. Sugawara E. Nikaido H. de Lorenzo V. Fernández L. A. (2002). Export of autotransported proteins proceeds through an oligomeric ring shaped by C-terminal domains. EMBO J. 21 (9), 2122–2131. 10.1093/emboj/21.9.2122 11980709 PMC125980

[B56] Wang R. Zhang Y. Ma Q. D. Y. Wu L. (2024). Recent advances of small molecule detection in nanopore sensing. Talanta 277, 126323. 10.1016/j.talanta.2024.126323 38810384

[B57] Watanabe H. Gubbiotti A. Chinappi M. Takai N. Tanaka K. Tsumoto K. (2017). Analysis of pore formation and protein translocation using large biological nanopores. Anal. Chem. 89 (21), 11269–11277. 10.1021/acs.analchem.7b01550 28980803

[B58] Waterhouse A. M. Procter J. B. Martin D. M. A. Clamp M. Barton G. J. (2009). Jalview Version 2—a multiple sequence alignment editor and analysis workbench. Bioinformatics 25 (9), 1189–1191. 10.1093/bioinformatics/btp033 19151095 PMC2672624

[B59] Wu E. L. Cheng X. Jo S. Rui H. Song K. C. Dávila-Contreras E. M. (2014). CHARMM-GUI membrane builder toward realistic biological membrane simulations. J. Comput. Chem. 35 (27), 1997–2004. 10.1002/jcc.23702 25130509 PMC4165794

[B60] Yoshimoto S. Ishii S. Kawashiri A. Matsushita T. Linke D. Göttig S. (2024). Adhesion preference of the sticky bacterium *Acinetobacter* sp. Tol 5. Front. Bioeng. Biotechnol. 12, 1342418. 10.3389/fbioe.2024.1342418 38375452 PMC10875045

[B61] Yoshimoto S. Sasahara J. Suzuki A. Kanie J. Koiwai K. Lupas A. N. (2025). Insights into the complex formation of a trimeric autotransporter adhesin with a peptidoglycan-binding periplasmic protein. Cell Surf. 14, 100155. 10.1016/j.tcsw.2025.100155 41103730 PMC12523085

[B62] Zhou W. Qiu H. Guo Y. Guo W. (2020). Molecular insights into distinct detection properties of α-hemolysin, MspA, CsgG, and aerolysin nanopore sensors. J. Phys. Chem. B 124 (9), 1611–1618. 10.1021/acs.jpcb.9b10702 32027510

[B63] Zimmermann L. Stephens A. Nam S.-Z. Rau D. Kübler J. Lozajic M. (2018). A completely reimplemented MPI bioinformatics toolkit with a new HHpred server at its core. J. Mol. Biol. 430 (15), 2237–2243. 10.1016/j.jmb.2017.12.007 29258817

