# Supplementary material for: Construction and characterization of a nanopore derived from the transmembrane domain of a trimeric autotransporter adhesin
Source: Front Bioeng Biotechnol. 2026 Feb 20;14:1764864. doi: 10.3389/fbioe.2026.1764864 (PMC12963341; doi:10.3389/fbioe.2026.1764864)
Supplement: Supplementary file 1 [file DataSheet1.pdf]

Supplementary Material

Construction and characterization of a nanopore derived from the transmembrane domain of a trimeric autotransporter adhesin

Jun Sasahara[1], Shogo Yoshimoto[1], Zugui Peng[2], Taehyun Hwang[1], Iori Kobayashi[1], Ryuji Kawano[2], Katsutoshi Hori[1]*

[1]Department of Biomolecular Engineering, Graduate School of Engineering, Nagoya University, Nagoya, Aichi 464-8603, Japan

[2]Department of Biotechnology and Life Science, Tokyo University of Agriculture and Technology (TUAT), Tokyo 184-8588, Japan

* Correspondence:
Katsutoshi Hori
Tel: +81-52-789-3339
E-mail: khori@chembio.nagoya-u.ac.jp

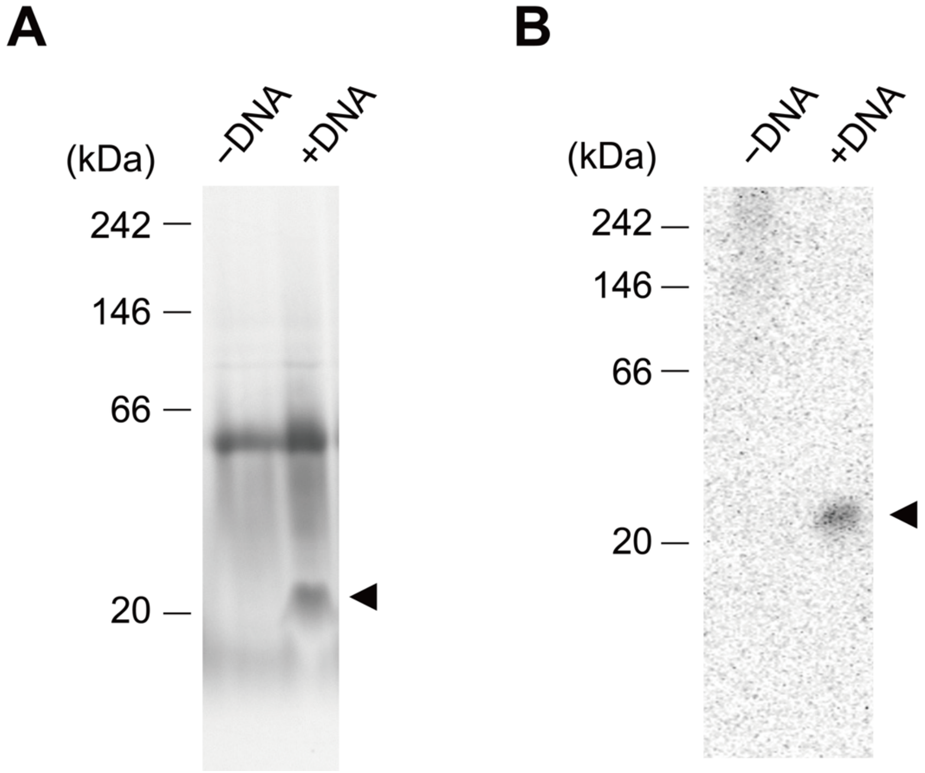

Supplementary Figure S1. Blue Native PAGE of AtaApore produced by a cell-free system. Total proteins from reactions lacking (-) or containing (+) the DNA encoding AtaApore were analyzed by Blue Native PAGE. The arrowhead indicates the band corresponding to the expected trimeric AtaApore complex. (A) CBB staining. (B) Western blotting using an anti-transmembrane domain antiserum.

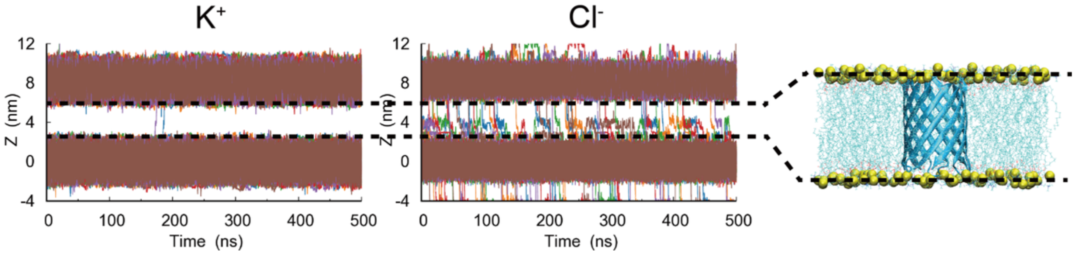

Supplementary Figure S2. Ion trajectories of AtaApore along the Z-axis during a 500 ns MD simulation. Overlay plots show positional changes of all K^+ and Cl^- ions along the Z-axis under a +150 mV transmembrane potential. Each trace represents an individual ion, shown in distinct colors for clarity. The right panel illustrates the simulation system containing AtaApore embedded in a lipid bilayer.

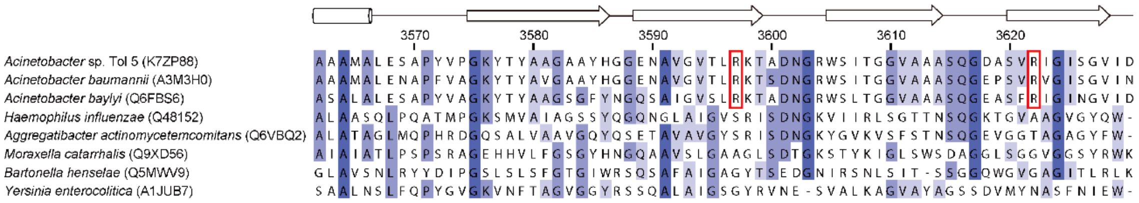

Supplementary Figure S3. Multiple sequence alignment of TAA transmembrane domains. Conserved arginine residues (R3597 and R3622) found in *Acinetobacter* are highlighted with red boxes.

Supplementary Note S1. DNA sequence of pUCFa-AtaApore.

The T7 promoter and Shine–Dalgarno (SD) sequence, start codon, His-tag, $AtaA_{3575–3630}$, stop codon, and T7 terminator.

GTAAAACGACGGCCAGTGAATTGTAATAGCTCTCTGAGTACGGAACACTCTTTCCCTTA
ACGACGCTCTTCCGATCTGATGAAATAATTTTGTTTAACTTTAAGAAGGAGATATACCA
ATGCATCATCATCATCATCATGGAAAATATACGTATGCAGCTGGTGCGGCTTACCACGG
CGGTGAAAACGCAGTCGGGGTTACCCTGCGTAAAACTGCCGACAACGGCCGCTGGTCTA
TCACCGGTGGCGTGGCTGCGGCATCCCAGGGTGATGCTAGCGTACGTATCGGCATTTCT
GGTGTTATCGACTAATGAATAACTAATCCTGAGCAATAACTAGCATAACCCCTTGGGGC
CTCTAAACGGGTCTTGAGGGGTTTTTTGATCAGATCGGAAGAGCACACGTCTGAACTCC
AGTCTTCAATGGTATCGCCCAGATAGCTTGGCGTAATCATGGTCATGGTCATAGCTGTT
TCCTGTGTGAAATTGTTATCCGCTCACAATTCCACACAACATACGAGCCGGAAGCATAA
AGTGTAAAGCCTGGGGTGCCTAATGAGTGAGCTAACTCACATTAATTGCGTTGCGCTCA
CTGCCCGCTTTCCAGTCGGGAAACCTGTCGTGCCAGCTGCATTAATGAATCGGCCAACG
CGCGGGGAGAGGCGGTTTGCGTATTGGGCGCTCTTCCGCTTCCTCGCTCACTGACTCGC
TGCGCTCGGTCGTTCGGCTGCGGCGAGCGGTATCAGCTCACTCAAAGGCGGTAATACGG
TTATCCACAGAATCAGGGGATAACGCAGGAAAGAACATGTGAGCAAAAGGCCAGCAAAA
GGCCAGGAACCGTAAAAAGGCCGCGTTGCTGGCGTTTTTCCATAGGCTCCGCCCCCCTG
ACGAGCATCACAAAAATCGACGCTCAAGTCAGAGGTGGCGAAACCCGACAGGACTATAA
AGATACCAGGCGTTTCCCCCTGGAAGCTCCCTCGTGCGCTCTCCTGTTCCGACCCTGCC
GCTTACCGGATACCTGTCCGCCTTTCTCCCTTCGGGAAGCGTGGCGCTTTCTCATAGCT
CACGCTGTAGGTATCTCAGTTCGGTGTAGGTCGTTCGCTCCAAGCTGGGCTGTGTGCAC
GAACCCCCCGTTCAGCCCGACCGCTGCGCCTTATCCGGTAACTATCGTCTTGAGTCCAA
CCCGGTAAGACACGACTTATCGCCACTGGCAGCAGCCACTGGTAACAGGATTAGCAGAG
CGAGGTATGTAGGCGGTGCTACAGAGTTCTTGAAGTGGTGGCCTAACTACGGCTACACT
AGAAGAACAGTATTTGGTATCTGCGCTCTGCTGAAGCCAGTTACCTTCGGAAAAAGAGT
TGGTAGCTCTTGATCCGGCAAACAAACCACCGCTGGTAGCGGTGGTTTTTTTGTTTGCA
AGCAGCAGATTACGCGCAGAAAAAAAGGATCTCAAGAAGATCCTTTGATCTTTTCTACG
GGGTCTGACGCTCAGTGGAACGAAAACTCACGTTAAGGGATTTTGGTCATGAGATTATC
AAAAAGGATCTTCACCTAGATCCTTTTAAATTAAAAATGAAGTTTTAAATCAATCTAAA
GTATATATGAGTAAACTTGGTCTGACAGTTACCAATGCTTAATCAGTGAGGCACCTATC
TCAGCGATCTGTCTATTTCGTTCATCCATAGTTGCCTGACTCCCCGTCGTGTAGATAAC
TACGATACGGGAGGGCTTACCATCTGGCCCCAGTGCTGCAATGATACCGCGAGACCCAC
GCTCACCGGCTCCAGATTTATCAGCAATAAACCAGCCAGCCGGAAGGGCCGAGCGCAGA
AGTGGTCCTGCAACTTTATCCGCCTCCATCCAGTCTATTAATTGTTGCCGGGAAGCTAG

AGTAAGTAGTTCGCCAGTTAATAGTTTGCGCAACGTTGTTGCCATTGCTACAGGCATCG
TGGTGTCACGCTCGTCGTTTGGTATGGCTTCATTCAGCTCCGGTTCCCAACGATCAAGG
CGAGTTACATGATCCCCCATGTTGTGCAAAAAAGCGGTTAGCTCCTTCGGTCCTCCGAT
CGTTGTCAGAAGTAAGTTGGCCGCAGTGTTATCACTCATGGTTATGGCAGCACTGCATA
ATTCTCTTACTGTCATGCCATCCGTAAGATGCTTTTCTGTGACTGGTGAGTACTCAACC
AAGTCATTCTGAGAATAGTGTATGCGGCGACCGAGTTGCTCTTGCCCGGCGTCAATACG
GGATAATACCGCGCCACATAGCAGAACTTTAAAAGTGCTCATCATTGGAAAACGTTCTT
CGGGGCGAAAACTCTCAAGGATCTTACCGCTGTTGAGATCCAGTTCGATGTAACCCACT
CGTGCACCCAACTGATCTTCAGCATCTTTTACTTTCACCAGCGTTTCTGGGTGAGCAAA
AACAGGAAGGCAAAATGCCGCAAAAAAGGGAATAAGGGCGACACGGAAATGTTGAATAC
TCATACTCTTCCTTTTTCAATATTATTGAAGCATTTATCAGGGTTATTGTCTCATGAGC
GGATACATATTTGAATGTATTTAGAAAAATAAACAAATAGGGGTTCCGCGCACATTTCC
CCGAAAAGTGCCACCTGACGTCTAAGAAACCATTATTATCATGACATTAACCTATAAAA
ATAGGCGTATCACGAGGCCCTTTCGTCTCGCGCGTTTCGGTGATGACGGTGAAAACCTC
TGACACATGCAGCTCCCGGAGACGGTCACAGCTTGTCTGTAAGCGGATGCCGGGAGCAG
ACAAGCCCGTCAGGGCGCGTCAGCGGGTGTTGGCGGGTGTCGGGGCTGGCTTAATTAAA
TGCCCCAAAACATCAGGTTAATGGCGTTCTTGATGTCATTCTCGCGATGCGACAGATCC
GCAACCTCTTCGCCAATTACCGACACTGGTACTGACGCCATATCCGTGGTCATCATACG
CCAGCTTTCATCCCCAATGTGGACAACCGGATAGAGTTCACGGGAAACTTTGTCGCTCA
GTAACCGTGCAGAAGCCAACGGAATCACCGTGCGACGACCAGGAGTATCGATGATGTCG
CTTTGCACATCCACAAACAGGCGATAGCGACTTTCGCGTTTGTAGGTGTAGACTTTGAA
CTGCGGTACGTTACAGAGAGCGCAACTGCTGTGACGGACGATCTGTACGCGCTTCTTCG
CTATTCCGCCAACTCGCAAACGGAGGATGTGCTGCAAGGCGATTAAGTTGGGTAACGCC
TGGGTTTTCCCAGTCACGACGTT

Supplementary Note S2. Amino acid sequence applied to AlphaFold2 structure prediction of AtaApore.

GKYTYAAGAAYHGGENAVGVTLRKTADNGRWSITGGVAAASQGDASVRIGISGVID

Supplementary Movie S1. Visualization of ion translocation through AtaApore.
The result of a 500 ns MD simulation is visualized. Cl^- ions are shown in yellow, and K^+ ions are shown in blue. Water molecules are omitted for clarity. Cl^- ions were observed to move through the pore in the negative direction along the z-axis. Although K^+ ion translocation events were rare, they can be seen at 0:57–0:58 s and 1:02–1:03 s, where K^+ ions move through AtaApore in the positive direction along the z-axis.

Supplementary Movie S2. Visualization of transient trapping of a Cl^- ion inside AtaApore.
A Cl^- ion is transiently trapped between residues R3597 and R3622 inside AtaApore. For clarity, only the trapped Cl^- ion is shown, while lipids and water molecules are omitted. Residues R3597 and R3622 are represented in the Licorice style in VMD.
